# Exposure to lead and dietary furan intake aggravates hypothalamus-pituitary-testicular axis toxicity in chronic experimental rats

**DOI:** 10.7555/JBR.36.20220108

**Published:** 2022-09-28

**Authors:** Solomon E. Owumi, Uche O. Arunsi, Moses T. Otunla, Imisioluwa O. Oluwasuji

**Affiliations:** 1 Cancer Research and Molecular Biology Laboratories, Department of Biochemistry, Faculty of Basic Medical Sciences, University of Ibadan, Ibadan, Oyo State 200004, Nigeria; 2 Department of Cancer Immunology and Biotechnology, School of Medicine, University of Nottingham, Nottingham NG7 2RD, UK

**Keywords:** infertility, furan, lead acetate, oxidative stress, DNA damage, inflammation

## Abstract

Lead (Pb) and furan are toxic agents, and persistent exposure may impair human and animal reproductive function. We therefore explored the effects of Pb and furan on male rat hypothalamic-pituitary-gonadal reproductive status, oxidative stress, inflammation, and genomic integrity. We found that co-exposure to Pb and furan reduced the activities of testicular function enzymes, endogenous antioxidant levels, total sulfhydryl group, and glutathione. Sperm abnormality, biomarkers of oxidative stress, inflammation, and p53 expression were increased in a dose-dependent manner by treatment with furan and Pb. Typical rat gonad histoarchitecture features were also damaged. Conclusively, co-exposure to Pb and furan induced male reproductive function derangement by decreasing the antioxidant defences in rats, increasing abnormalities in spermatozoa morphology, and reducing reproductive hormone in circulation. These pathophysiological alterations, if persistent, might provide a permissive environment for potentiating reproductive dysfunction and infertility.

## Introduction

The factors that promote infertility include socioeconomic and behavioural characteristics, tobacco smoking, insulin resistance, sleep apnoea, reduced level of gonadotropins and testosterone, and underlying medical conditions^[1–2]^. Recent findings revealed that exposure to environmental factors such as heavy metals—lead (Pb), cadmium, chromium, manganese, mercury, and nickel; and furan, a by-product of food processing involving heat treatment, can predispose men to infertility^[3]^.

Pb is a heavy metal known to impact spermatogenesis^[4]^, and can cause reproductive dysfunction^[5]^. Environmental pollution from Pb can occur from mining activities, Pb-based gasoline, pipe, ammunition, vehicle exhaust, electronic wastes, erosion of natural wastes, re-processing Pb-battery, natural fires, and sea salt spray^[6]^. When Pb enters the body, either through ingestion of contaminated feed or water, it is distributed to two chief compartments depending on whether the exposure is acute or chronic. Acute Pb exposure is distributed to readily exchangeable pools including the blood, liver, kidney, and brain, while chronic exposure will accumulate Pb in deep tissue reservoirs, including the bones^[7]^. The World Health Organization (WHO) and the United States Centre for Disease Control and Prevention (CDC) set the permissible blood level of Pb (<10 μg/dL in adults and 5 μg/dL in children)^[8]^. Despite these reference values, Pb toxicities have roused concerns globally. The toxicological impacts of Pb exposure have been investigated at low and high concentrations. Some studies report that the blood concentration of Pb in adults (>100 μg/dL) and children (approximately 80–100 μg/dL) can cause cognitive impairment^[9]^. A minimal concentration below 3 μg/dL of Pb, caused cognitive deterioration and maladaptive behaviour in humans and animals^[10]^, implying that there is no acceptable baseline for Pb exposure. Also, exposure to Pb has been attributed to reproductive health debility^[11]^. A rise in seminal plasma or blood Pb levels (<10 μg/dL, 53 μg/dL, 40 μg/dL, and <15 μg/dL) negatively correlate with reductions in sperm count and density, decreased ejaculate volume, immature and abnormal spermatozoa, and drop in total sperm count of exposed individuals^[12]^, respectively.

Furan is a by-product of food processing and preservation technique involving heat treatment including cooking, roasting, baking, pasteurisation, and sterilisation. Formation of furan occurs by Maillard reaction; a non-enzymatic response of amino acids, peptides, and proteins with reducing sugars and vitamin C^[13]^. In a 2011 report, the European Food Safety Authority (EFSA) detected a high level of furan in coffee brands, including roasted bean coffee (3660 ng/g), unspecified coffee (2016 ng/g), roasted ground coffee (1936 ng/g), and instant coffee (394 ng/g). Also, low levels of furan were detected in food products such as soft drinks (0.8–1.2 ng/g), tea (1–1.7 ng/g), wine and liquors (1.3 ng/g), vegetable fats (1.5–1.7 ng/g), and fruit juice (2.2–4.6 ng/g)^[14]^. Furan toxicity is mediated by cytochrome P450 2E1 involved in furan metabolism, to the reactive intermediate, *cis*-but-ene-1,4-dialdehyde (BDA). BDA interacts with amino acids, proteins, and DNA to induce toxicities in significant organs of the body, thus making furan a global public health issue. The foregoing necessitated the European Commission to ask the EFSA Panel on Contaminants in the Food Chain to conduct a scientific evaluation on the risk to humans of furan in foods. The calculated margin of exposure obtained by the EFSA indicated a health concern^[15]^. More worrisome is the documentaries from the US Food and Drug Administration (US FDA), EFSA, National Toxicology Program (NTP), and International Agency for Research on Cancer (IARC) that furan is carcinogenic to humans^[13]^.

In developing countries, exposure of humans and experimental animals to Pb and furan may emanate from antiquated facilities for transporting municipal water from pump stations to residential homes. Increases in anthropogenic activities including Pb-ore processing, Pb-battery recycling, ammunition, vehicle exhaust, e-wastes and bush burning, and poor-quality control practices on the preservation of food products also contribute to these exposure routes^[13]^. Hence, there is a need to monitor the degree of toxicities following combined exposure to Pb and furan since humans and animals quickly ingest them through their diets and drinking water. A feasible means of investigating the harmful effects of environmental toxicants in experimental models is to monitor the process of oxidative stress-mediated by increased production of reactive oxygen species (ROS) with a concurrent decline in endogenous antioxidants, signalling in favour of pro-inflammation mediators and loss of genomic integrity. We hypothesised against this background that accidental exposure to Pb and furan may significantly impair male rats' reproductive health. Therefore, the present study was aimed at investigating the combined effects of Pb and furan on the integrity and functionality of the hypothalamic-pituitary-testicular axis in rats by examining sperm functionality, testicular function biomarkers, reproductive hormones, genomic stability, and oxido-inflammatory biomarker in rats. We observed that co-exposure to Pb and furan altered hypothalamic-pituitary-testicular system function by primarily exacerbating oxidative-inflammatory stressors, and genomic instability.

## Materials and methods

### Chemicals and reagents

Lead acetate, furan, epinephrine, glutathione (GSH), thiobarbituric acid, hydrogen peroxide (H_2_O_2_), 5,5-dithio-bis-2-nitrobenzoic acid, Griess reagent, 1-chloro-2,4-dinitrobenzene (CDNB), trichloroacetic acid, and bovine serum albumin were purchased from Sigma (USA). Disodium hydrogen phosphate, sodium carbonate, sodium hydroxide, sodium-potassium tartrate, and sodium chloride were obtained from BDH Ltd. (UK) and William Hopkins Ltd. (UK). Acid phosphatase, alkaline phosphatase (ALP), lactate dehydrogenase (LDH) kits were obtained from Randox Laboratories Ltd. (UK). Enzyme-linked immunosorbent assay (ELISA) kits for the assessment of p53 and tumour necrosis factor-alpha (TNF-α) were obtained from Elabscience Biotechnology (China), and luteinizing hormone, follicle-stimulating hormone, prolactin, and testosterone kits from Fortress Diagnostics (UK). All other chemicals used for these experiments are of analytical grade.

### Sample size estimation and care of animals

The 3Rs (replacement, reduction, and refinement) guidelines for care and use of experimental animals were adopted in this study as previously described^[2]^. The sample size was estimated with the G* Power software (v3.1.9.4)^[16]^ (University of Dusseldorf, Germany). To avoid type Ⅰ and Ⅱ errors and an effect size of 0.40 (larger effect) at 0.05 alpha error of probability for one-way analysis of variance (ANOVA) was employed, giving a total sample size of 125 at 95% power. Out of 125 estimated experimental animals, 30 (six groups; *n*=5 per group) male Albino Wistar rats (approximately 177 g body weight [BW]) were purchased and housed in the Department of Biochemistry University of Ibadan. The rats were acclimatised for seven days under natural daily photoperiod; fed and provided clean water* ad libitum*.

### Experimental outline

**Figure 1:**
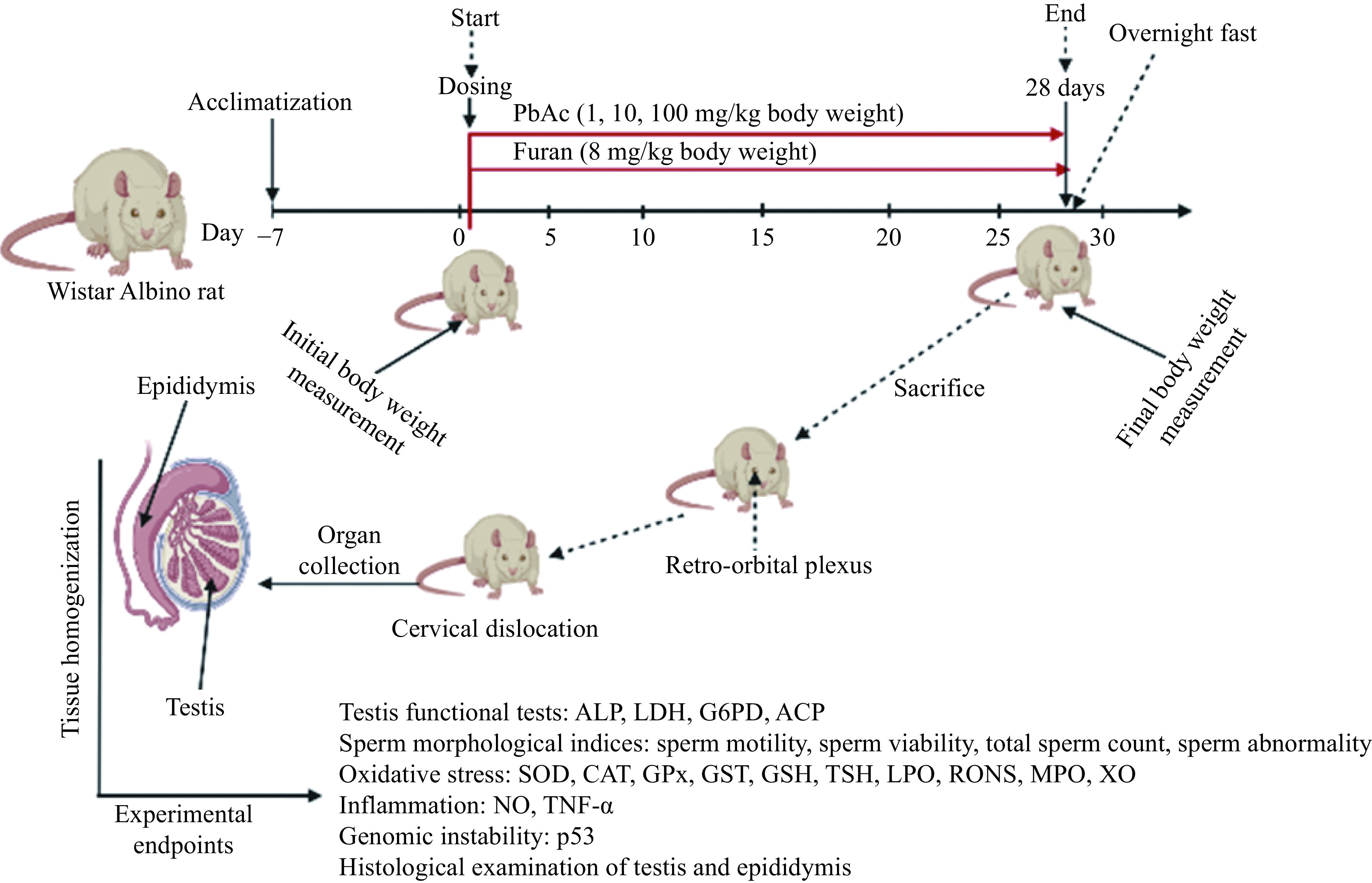
Experimental protocol of lead acetate and furan treatments on the hypothalamus-pituitary-testicular system in male Albino Wistar rats treated for 28 consecutive days.

Treatment of the rats for 28 successive days was initiated following the study's approval by the University of Ibadan Animal Care and Use Research Ethics Committee (Approval No. UI-ACUREC/032-0525/27). The furan stock solution (8 mg/kg)^[17]^ was prepared by dissolving 213 μL furan in corn oil and administered *per os* (*p.o.*) according to the BW of rats. PbAc stock solution (0.1 mg/mL)^[18]^ was prepared with 50 mg PbAc in 50 mL of distilled water. Subsequently, 1, 10, and 100 μg/L were prepared by adding 0.02, 0.2, and 2 mL of PbAc stock and reconstituted to 2 L with distilled water. Experimental rats water intake was estimated daily, and replenished to maintain a daily supply of 300 mL. Rats in the control group received corn oil, the furan group received furan (8 mg/kg BW) *p.o.*, the PbAc group received PbAc (100 μg/kg BW) in drinking water* p.o*, the furan+PbAc_1_ group received furan (8 mg/kg BW) and PbAc (1 μg/kg BW), the furan+PbAc_2_ group received furan (8 mg/kg BW) and PbAc (10 μg/kg BW), and the furan+PbAc_3_ group received furan (8 mg/kg) and PbAc (100 μg/kg BW) (***Fig. 1***).

### Experimental endpoints and tissue and organ harvest

After 28-days of treatment, the rats were fasted (24 h), whole blood collected into non-heparinised tubes *via* retro-orbital venous plexus, and subsequently sacrificed by cervical displacement^[19]^. The blood was left to coagulate and then centrifuged (10 min; 1000 *g*) to obtain the serum for sex hormones estimation. The testis, epididymis, and hypothalamus were immediately excised, weighed and processed for biochemical analysis and histopathology. The relative weight (%) of the organ (*e.g.*, hypothalamus, testis, and epididymis) were calculated accordingly: (weight of the organ)/(weight of the body)×100.

Using a Teflon homogeniser, the organ samples used for biochemical assays were prepared by homogenising in 0.1 mol/L of phosphate buffer at pH 7.4. The homogenates were cold centrifuged (15 min; 20 000 *g*) to obtain a clear supernatant (mitochondria fraction) collected and frozen in aliquots pending biochemical analysis.

### Analysis of sperm functional parameters

The testicular contents of the rats after treatment were subjected to further analysis to determine sperm motility as previously described by Zemjanis^[20]^. Epididymal sperm number was determined according to the WHO protocol^[21]^. Sperm viability and morphological defects were measured according to the method of Wells and Awa^[22]^.

### Evaluation of reproductive hormone levels in the serum

After the treatment, the serum of rats was assayed for luteinising hormone (LH), follicle-stimulating hormone (FSH), prolactin, and testosterone level using ELISA kits according to the protocols of the manufacturers. The data were acquired with a Microplate Reader Spectra Max 340 (Molecular Devices, USA).

### Determination of testicular enzyme biomarkers and biomarkers of oxidative stress, inflammation and DNA perturbation

The tissue homogenate of the rats after treatment was subjected to biochemical analysis, and total protein concentrations were determined by Bradford's method^[23]^. Activities of testicular function biomarkers—acid and alkaline phosphatases (ACP and ALP)—were assessed by the method as reported^[24–25]^. Glucose-6-phosphate dehydrogenase (G6PD) was determined by Wolf *et al* method^[26]^. Also, lactate dehydrogenase-X was assessed by Vassault method^[27]^. Biomarkers of oxidative stress including the activity of superoxide dismutase (SOD) was evaluated by the method of Misra and Fridovich^[28]^; total sulfhydryl (TSH) concentration was evaluated by Ellman process^[29]^; catalase (CAT) activity was measured according to the protocols of Clairborne using H_2_O_2_ as a substrate as previously reported^[30]^; glutathione-S-transferase (GST) and glutathione peroxidase (GPx) were estimated by the methods of Habig^[31]^ and Rotruck *et al*^[32]^, respectively. Reduced GSH was determined following Jollow *et al* method^[33]^, while xanthine oxidase (XO) was quantified by Bergmeyer *et al* method^[34]^. Also, malondialdehyde (MDA), a biomarker of lipid peroxidation, was quantified by the method of Okhawa and Yagi^[35]^, and reactive oxygen and nitrogen species (RONS) concentration was measured by the method of Owumi and Dim^[36]^. Furthermore, biomarkers of inflammation, including nitric oxide (NO) and myeloperoxidase (MPO), were estimated by the protocols of Green *et al*^[37]^ and Granell *et al*^[38]^, respectively. TNF-α and a biomarker of genomic integrity (p53) were estimated by ELISA kits according to the manufacturer's protocol.

### Histological assessment

Microscopic assessment of the testes was performed following standard dehydration and paraffin embedding method^[39]^, and haematoxylin and eosin stained tissue slides prepared. The prepared slides were coded and examined under a Carl Zeiss microscope (Germany). On inspection, images were taken using a Zeiss Axiocam 512 camera by a pathologist unaware of the various treatment groups from which the slides were prepared.

### Statistical analysis

Paired Student's *t*-tests were used to analyse the data generated to determine the significance level of the mean body weight of rats before and after treatment. One-way analysis of variance (ANOVA) followed by Tukey's *post hoc* test was used to determine the significant difference across the different experimental cohorts (GraphPad Prism version 8.3.0 for Mac; GraphPad, USA). The results are expressed as means±SD of replicates, and statistically significant differences were set at a value of *P*<0.05.

## Results

### Effects of combined treatment of Pb and furan on mean body weight, organ weight, and water intake of rats

**Figure 2:**
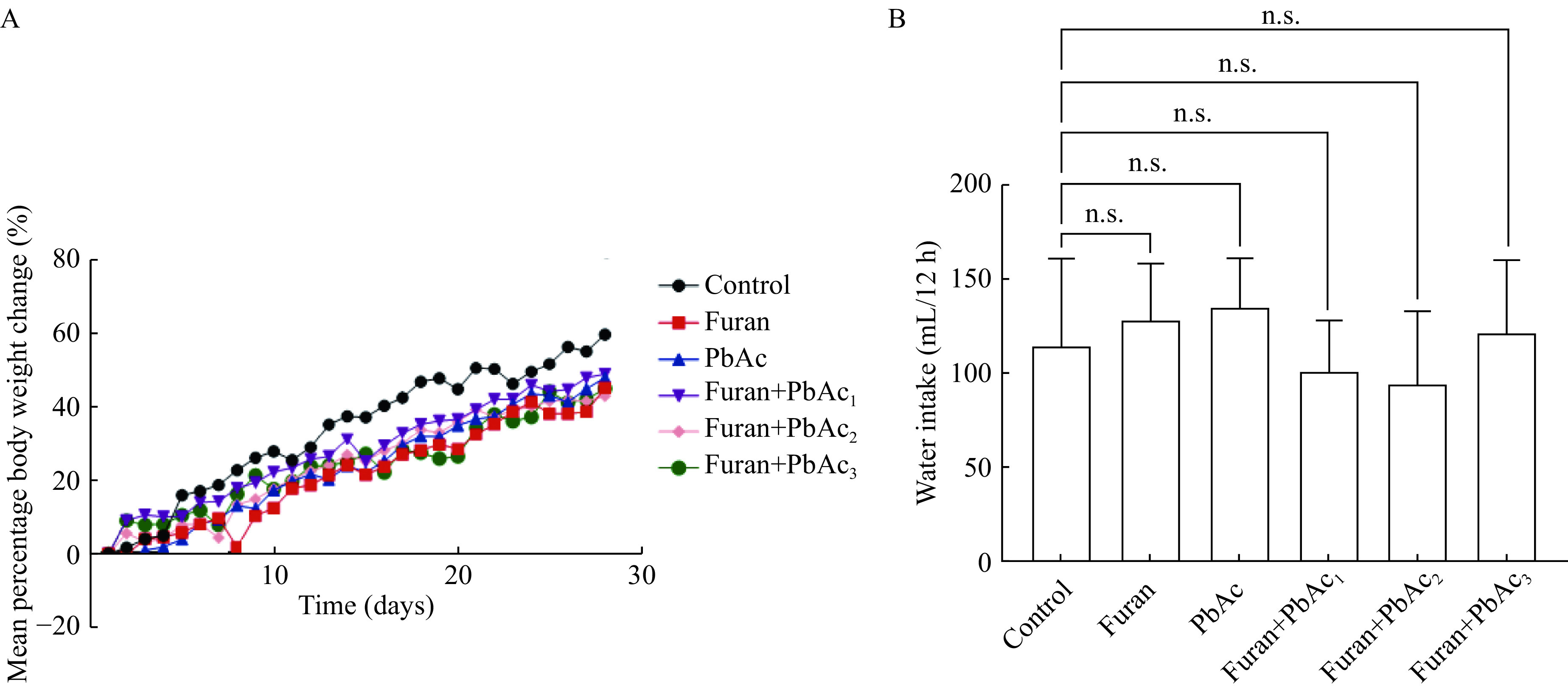
PbAc and furan treatments lowered mean body weight and water intake in male Albino Wistar rats.

The effects of Pb and furan on the hypothalamic-pituitary-gonadal axis were investigated using male Wistar Albino rats. PbAc and furan were administered to rats for 28 consecutive days and the changes in mean body weight, organ weight, and water intake were examined. It was revealed that treatments with PbAc and furan slightly waned (*P*>0.05) the mean percentage body weight change of rats (***Fig. 2A***). In addition, PbAc and furan treatments slightly decreased (*P*<0.05) the mean body weight of the animals compared to the control in the order: furan+PbAc_3_ < furan+PbAc_2_ < furan < furan+PbAc_1_ < PbAc < control, although this trend was not statistically significant within the exposure time of 28 days. Similarly, PbAc and furan treatment slightly altered the mean organ weight and relative organ weight of the hypothalamus, testis, and epididymis relative to the control (***Table 1***). The result further revealed that the water intake in the control group was not affected but was altered in groups treated with furan and PbAc (***Fig. 2B***). The results indicated that PbAc and furan slightly altered the mean body weight and organosomatic indices of the experimental rats post 28 days exposure.

**Table 1:** Effect of combined treatment of furan and lead acetate on body weight, organ weight and relative organ weight of rats

Characteristics	Control	Furan	PbAc	Furan+PbAc_1_	Furan+PbAc_2_	Furan+PbAc_3_
IBW (g)	152.60±11.24	189.00±10.34	193.60±11.15	180.40±9.71	169.80±4.15	174.60±3.58
FBW (g)	231.75±18.80^#^	257.00±7.00^#^	265.80±8.53^#^	249.40±15.47^#^	229.00±14.78^#^	233.50±12.50^#^
Testis weight (g)	2.48±0.23	2.76±0.22	2.56±0.26	2.64±0.17	2.36±0.39	2.68±0.23
Epididymis weight (g)	0.35±0.06	0.34±0.04	0.39±0.09	0.51±0.13	0.58±0.09	0.51±0.21
Hypothalamus weight (g)	0.04±0.02	0.05±0.00^*^	0.07±0.01	0.04±0.01	0.05±0.00	0.05±0.03
RTW (%)	1.11±0.11	1.06±0.10	0.96±0.08	1.06±0.10	1.03±0.13	1.16±0.08
REW (%)	0.16±0.02	0.13±0.01	0.15±0.03	0.21±0.06	0.26±0.05^*^	0.22±0.08
RHW (%)	0.02±0.01	0.02±0.00	0.03±0.00	0.02±0.00	0.02±0.00	0.02±0.01
Rats were administered with furan alone (8 mg/kg BW), PbAc alone (100 μg/kg BW), furan (8 mg/kg BW) plus PbAc (1 μg/kg BW [PbAc_1_], 10 μg/kg BW [PbAc_2_], 100 μg/kg BW [PbAc_3_]), or vehicle for 28 consecutive days of feeding (*n*=5 per group). Data are expressed as mean±SD. Differences between the treatment groups were analyzed by Student's *t*-test. ^#^*P<*0.05 versus IBW; ^*^P<0.05 versus control. PbAc: lead acetate; IBW: initial body weight; FBW: final body weight; RTW: relative testis weight; REW: relative epididymis weight; RHW: relative hypothalamus weight.						

### Effects of combined treatment of Pb and furan on reproductive function and integrity of rats

**Figure 3:**
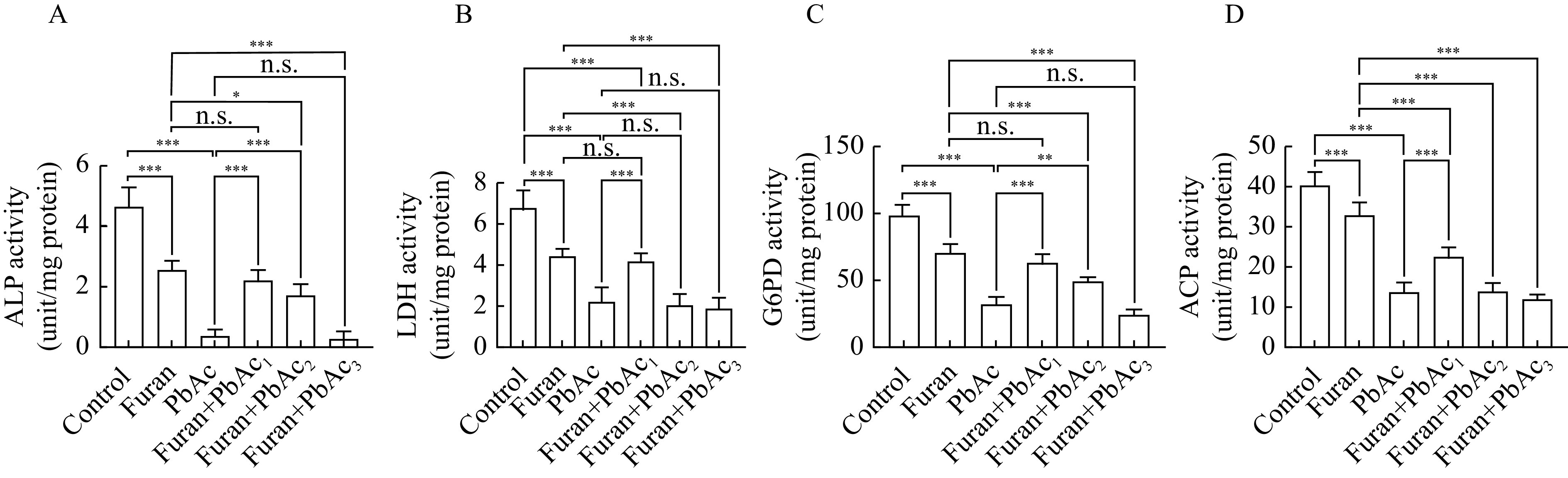
Lead and furan treatments lowered the activities of testicular enzymes in male rats.

The effects of Pb and furan on reproductive function and integrity of rats were examined in this study. Both individual exposure to furan or PbAc and combined exposure to furan and PbAc led to significantly decreased (*P*<0.05) percentages of sperm motility and viability, as well as sperm count compared to the control group. Conversely, sperm abnormality indices, including abnormality of the head (%), irregularity of the mid-piece (%), abnormality of the tail (%), and total abnormality (%) were significantly increased (*P*<0.05) in groups treated with furan and/or PbAc compared to the control (***Table 2***). In addition, the activities of ALP, LDH, and G6PD in cohorts of rats treated with furan and PbAc at 10 and 100 μg/kg BW were significantly waned (*P*<0.05) compared to furan alone group, while the activity of ACP in cohorts of rats treated with furan and PbAc at 1, 10 and 100 μg/kg BW were significantly reduced (*P*<0.05) compared to furan alone group (***Fig. 3***).

**Table 2:** Combined effects of furan and lead acetate on the sperm morphological characteristics of rats

Characteristics	Control	Furan	PbAc	Furan+PbAc_1_	Furan+PbAc_2_	Furan+PbAc_3_
Sperm functional characteristics						
Motile sperm (%)	92.00±2.73	72.00±4.47^*^	72.00±4.47^*^	70.00±7.07^*^	64.00±5.47^*^	62.00±4.47^*^
Viable sperm (%)	97.40±1.34	78.00±5.70^*^	71.00±4.18^*^	76.00±6.51^*^	69.00±4.18^*^	60.00±7.91^*^
Sperm count (10^7^/epididymis)	129.00±10.15	102.60±4.50^*^	95.40±10.21^*^	98.40±6.35^*^	94.80±5.76^*^	85.00±4.18^*^
Sperm abnormality						
Abnormality of the head (%)	1.85±0.23	2.23±0.34	2.80±0.17^*^	2.24±0.20	2.59±0.37^*^	2.90±0.14^*^
Abnormality of the mid-piece (%)	3.06±0.21	4.37±0.22^*^	5.09±0.33^*^	4.86±0.14^*^	4.86±0.51^*^	5.70±0.19^*^
Abnormality of the tail (%)	3.53±0.48	5.86±0.32^*^	6.21±0.61^*^	4.85±0.75^*^	5.86±0.76^*^	6.80±0.34^*^
Total abnormality (%)	8.43±0.47	23.46±0.59^*^	14.10±0.66^*^	11.95±0.90^*^	13.31±0.74^*^	15.40±0.41^*^
Rats were administered with furan alone (8 mg/kg BW), PbAc alone (100 μg/kg BW), furan (8 mg/kg BW) plus PbAc (1 μg/kg BW [PbAc_1_], 10 μg/kg BW [PbAc_2_], 100 μg/kg BW [PbAc_3_]), or vehicle for 28 consecutive days of feeding (*n*=5 per group). Data are expressed as mean±SD. Differences between the treatment groups were analyzed by Student's *t*-test. ^*^P<0.05 versus control. PbAc: lead acetate.						

**Figure 4:**
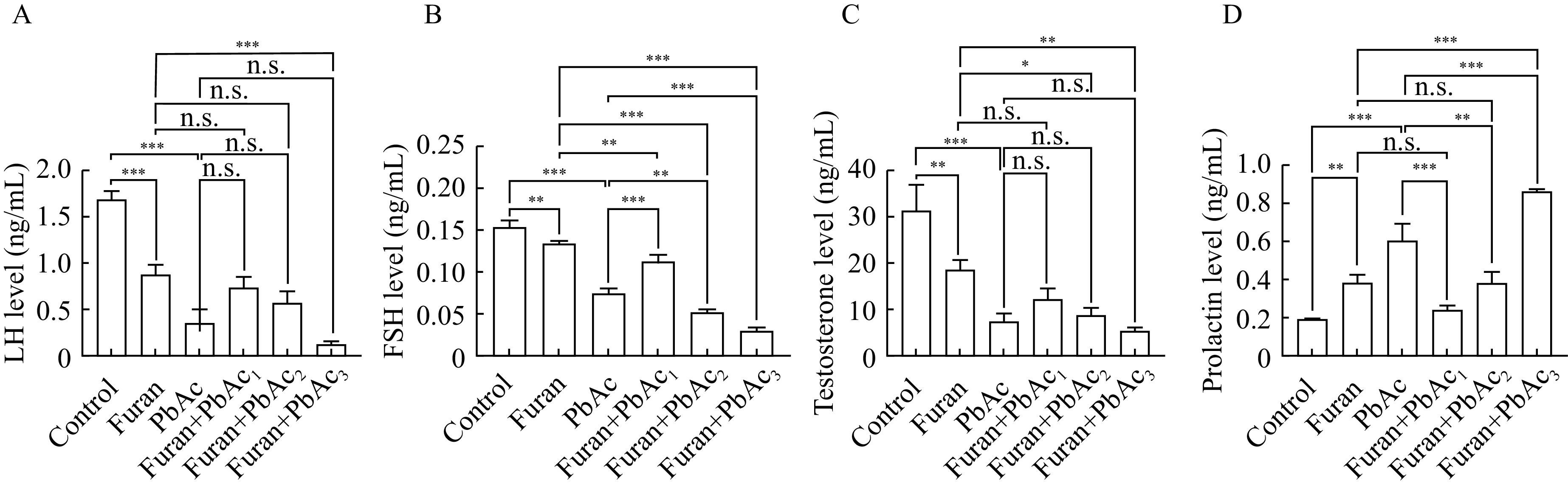
Lead and furan treatments impaired the steroidogenic hormone levels in male Albino Wistar rats.

Furthermore, alterations in the basal level of gonadotropin-releasing hormones such as LH and FSH, as well as other reproductive hormones such as testosterone and prolactin, were observed in this study. The results revealed that treatments with PbAc and furan, either individually or in combinations, significantly decreased (*P<*0.05) the levels of LH, FSH, and testosterone while significantly increasing (*P*<0.05) the level of prolactin (an indication of reproductive toxicity). Compared to furan alone group, the level of LH was significantly (*P*<0.05) lowered in group treated with furan and PbAc at 100 μg/kg BW; the level of FSH was significantly (*P*<0.05) reduced in groups treated with furan and PbAc at 1, 10, and 100 μg/kg BW, while the levels of testosterone and prolactin were significantly (*P*<0.05) altered in groups treated with furan and PbAc at 10 and 100 μg/kg BW (***Fig. 4***). Taken together, the above results indicated that the administration of PbAc and furan synergistically altered the integrity and functionality of the reproductive system of male rats.

### Effects of combined treatment of Pb and furan on redox balance of rats

**Figure 5:**
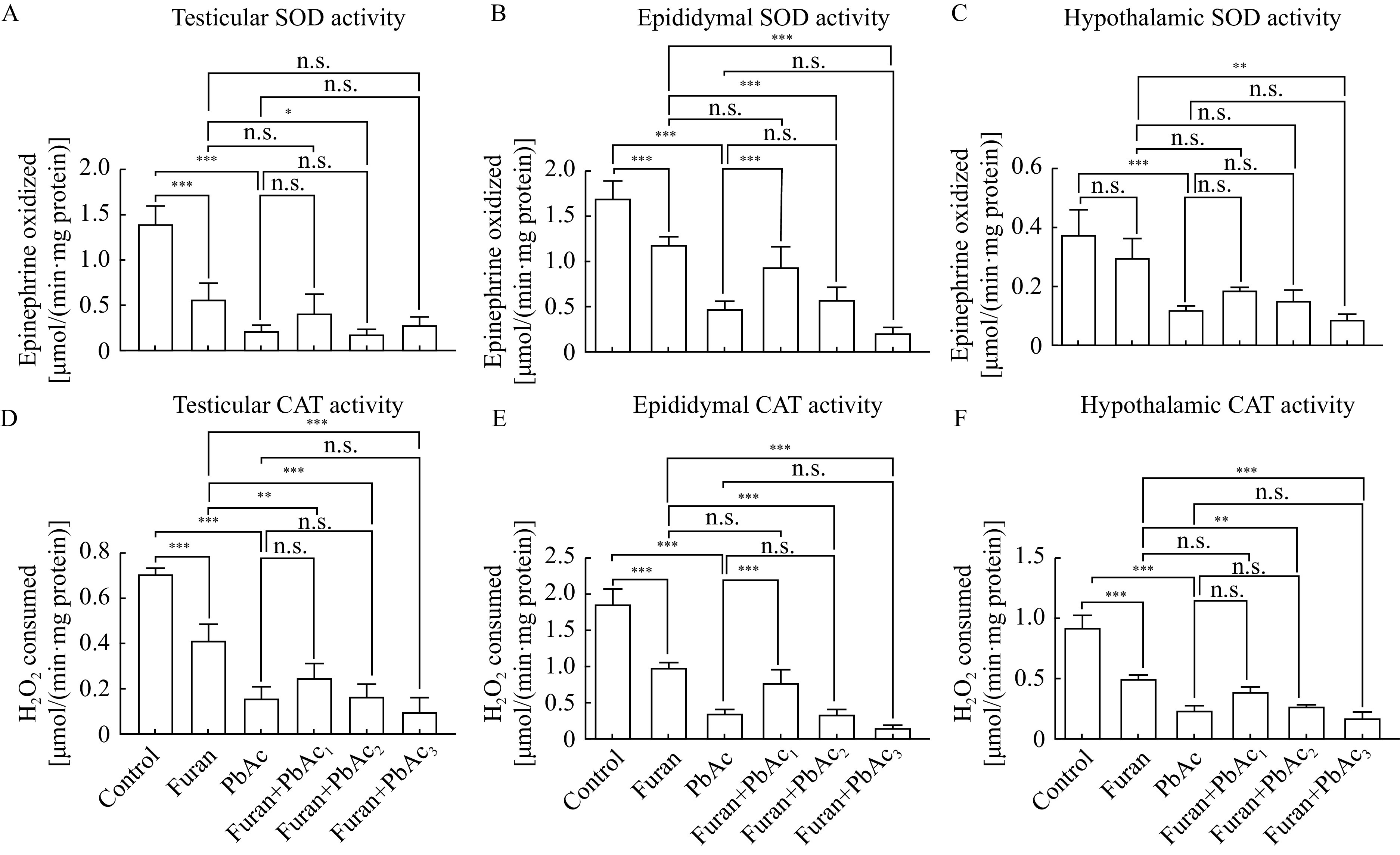
Lead and furan treatments waned the activities of endogenous SOD and CAT in male Albino Wistar rats.

**Figure 6:**
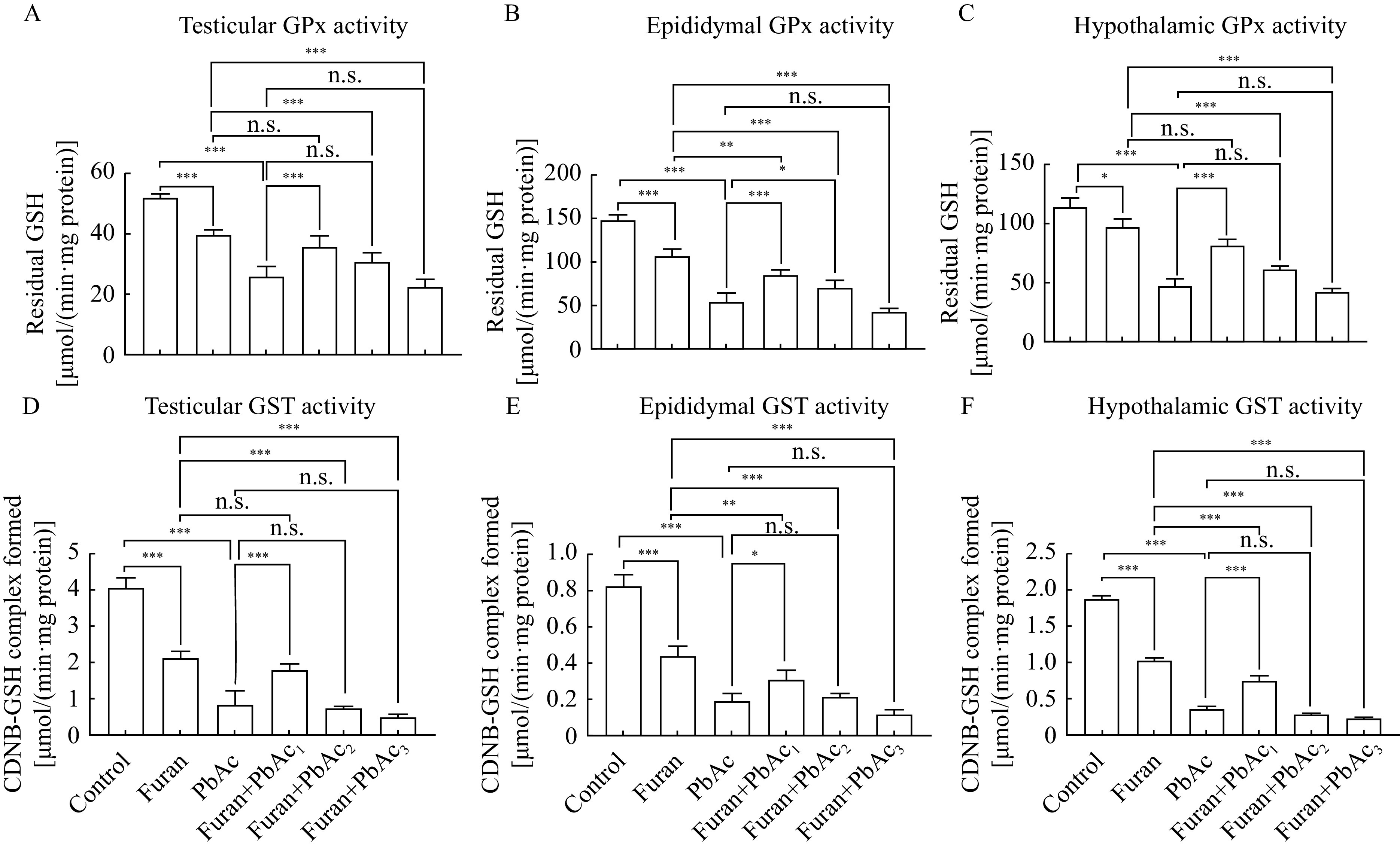
Lead and furan treatments waned the activities of endogenous GPx and GST in male Albino Wistar rats.

**Figure 7:**
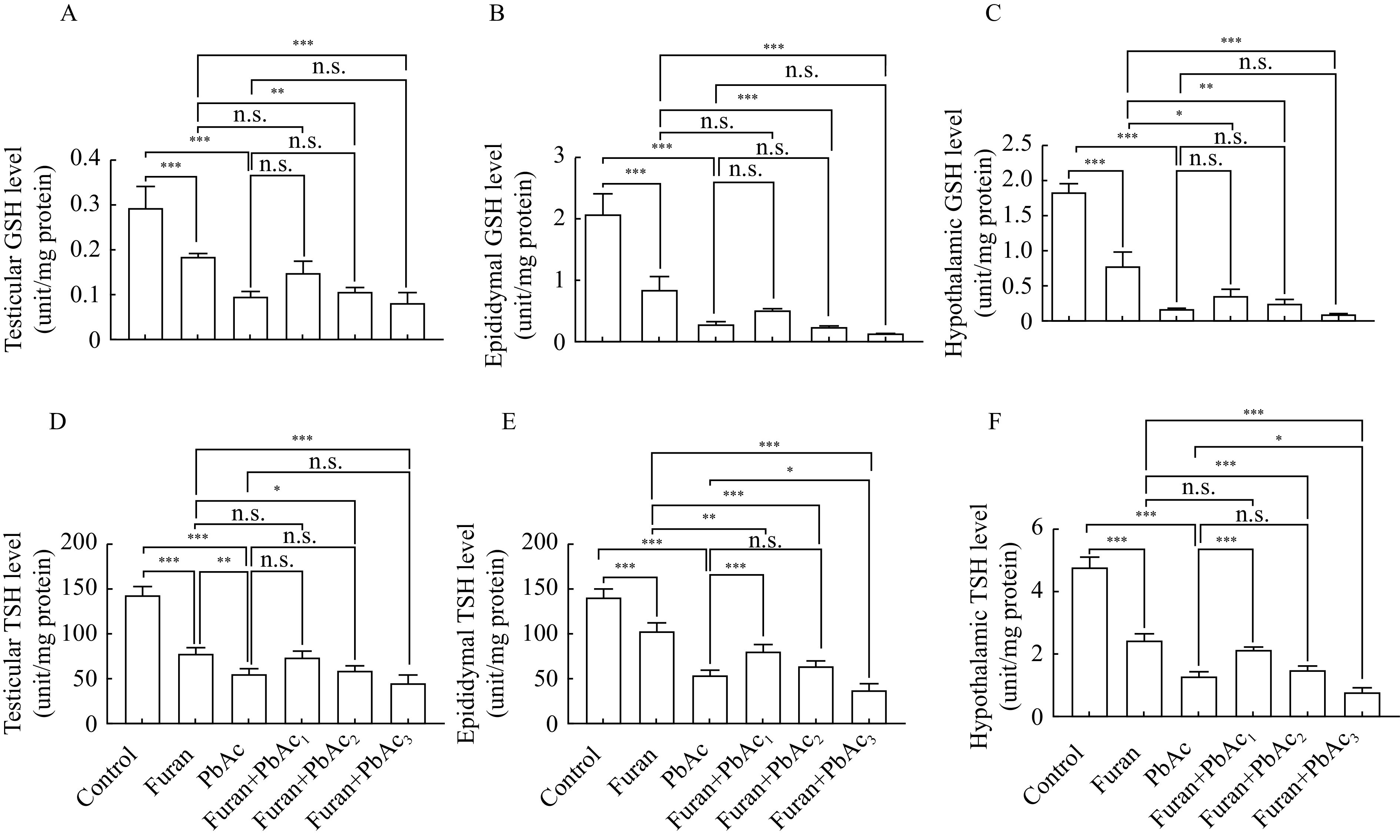
Lead and furan treatments waned the levels of endogenous GSH and TSH in male Albino Wistar rats.

**Figure 8:**
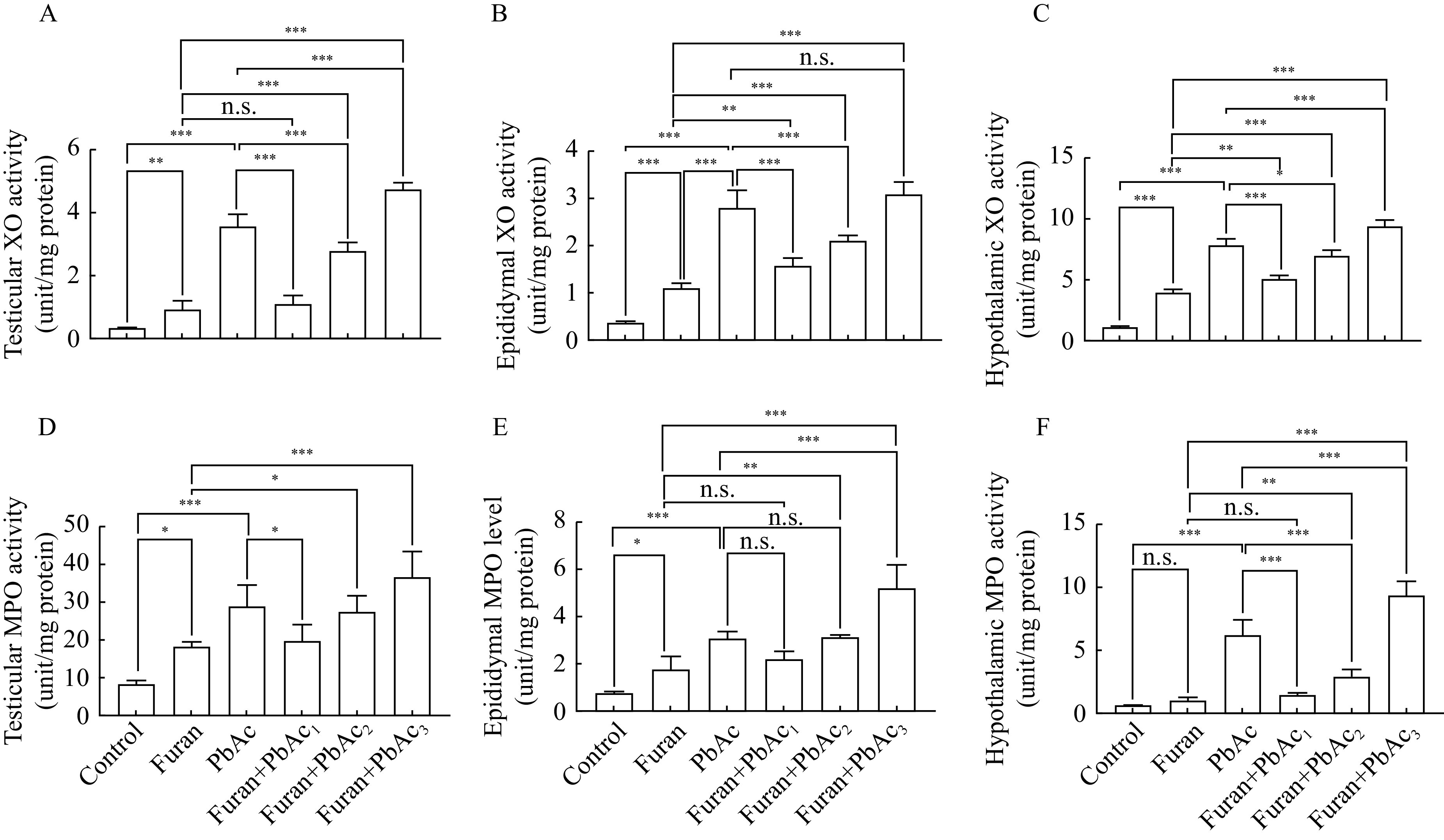
Lead and furan treatments elevated the activities of XO and MPO in male Albino Wistar rats.

**Figure 9:**
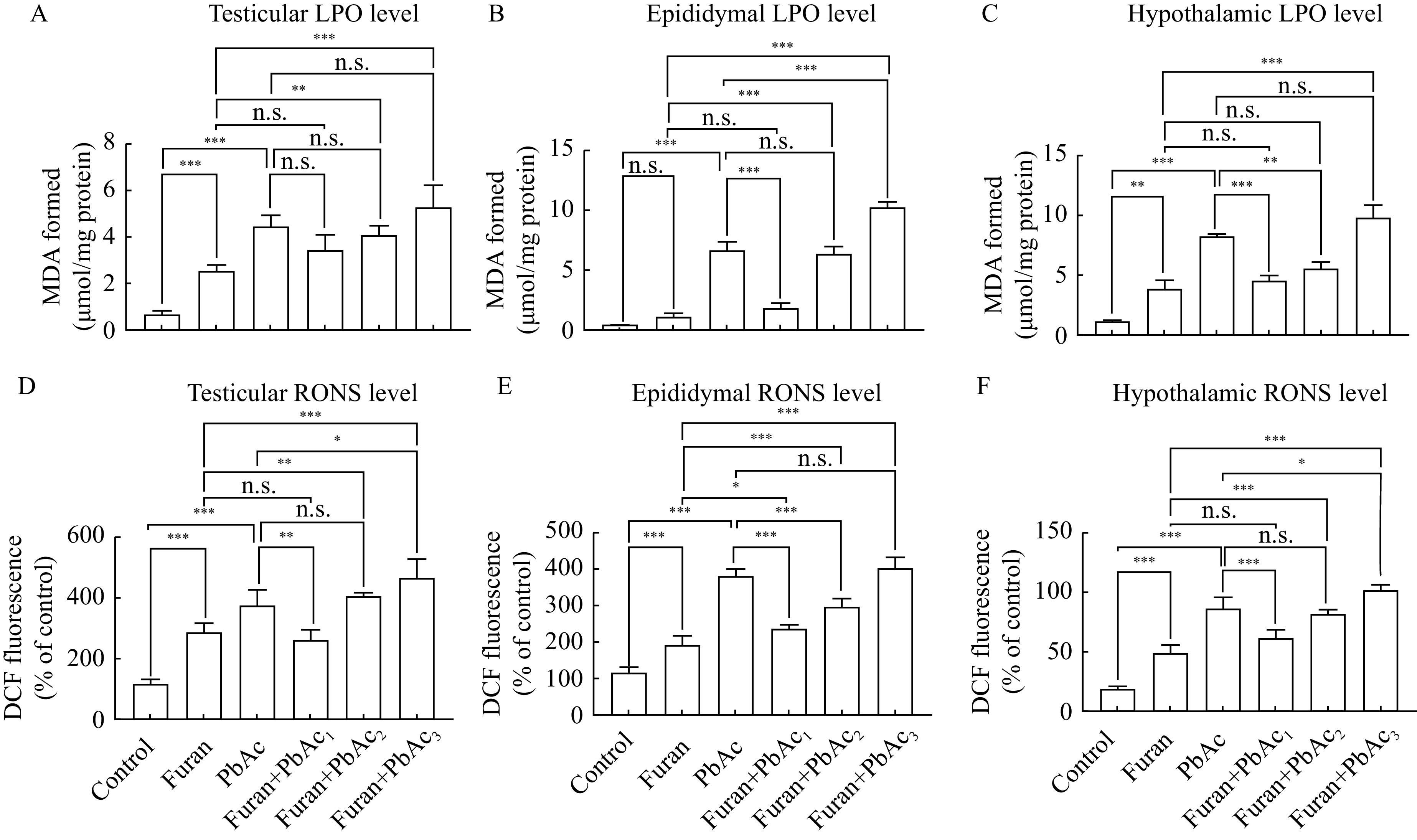
Lead and furan treatments elevated the levels of LPO and RONS in male Albino Wistar rats.

The impacts of PbAc and furan treatment on the redox balance of the hypothalamic-pituitary-gonadal axis of male rats were evaluated. Exposure of Pb and/or furan significantly decreased (*P<*0.05) the levels of SOD, CAT, GPx, GST, GSH, and TSH in the hypothalamus, testis, and epididymis of male rats compared to the control group rats. This effect was further potentiated (*P<*0.05) in cohorts of rats co-treated with furan and Pb at multiple concentrations (***Fig. 5***–***7***). Compared to furan alone group, the activities of SOD were significantly (*P<*0.05) decreased in groups treated with furan and PbAc at 10 μg/kg BW in the testis; furan and PbAc at 10 and 100 μg/kg BW in the epididymis, and furan and PbAc at 100 μg/kg BW in the hypothalamus. In addition, the activities of CAT were significantly (*P<*0.05) waned in groups treated with furan and PbAc at 1, 10, and 100 μg/kg BW in the testis; furan and PbAc at 10 and 100 μg/kg BW in the epididymis and hypothalamus compared to furan alone group (***Fig. 5***). The activities of GPx in cohort of rats were significantly (*P<*0.05) lowered in groups treated with furan and PbAc at 10 and 100 μg/kg BW in the testis, epididymis and hypothalamus compared to group treated only with furan. The results further divulged that the activities of GST were also significantly (*P<*0.05) diminished in groups treated with furan and PbAc at 10, and 100 μg/kg BW in the testis, epididymis and hypothalamus compared to furan alone group (***Fig. 6***). The levels of reduced-GSH were significantly (*P<*0.05) decreased in groups treated with furan and PbAc at 10 and 100 μg/kg BW in the testis and epididymis and PbAc at 1, 10, and 100 μg/kg BW in the hypothalamus compared to group treated only with furan. In addition, the results revealed that the activities of TSH were significantly (*P<*0.05) reduced in groups treated with furan and PbAc at 10, and 100 μg/kg BW in the testis and hypothalamus, and PbAc at 1, 10, and 100 μg/kg BW in the epididymis compared to furan alone group (***Fig. 7***). In comparison to the control, we observed that treatment with Pb and furan significantly increased the tissue levels of XO, MPO, LPO, and RONS (*P<*0.05). The activities and levels of these oxidative mediators were further increased as the concentration of Pb was raised (from 1 to 100 μg/L at a constant value of furan [8 mg/kg BW]) (***Fig. 8*** and ***9***). The results implied that PbAc and furan treatments impaired the redox system along the hypothalamic-pituitary-gonadal axis in male rats.

### Effects of combined treatment of Pb and furan on inflammatory response and genomic integrity of rats

**Figure 10:**
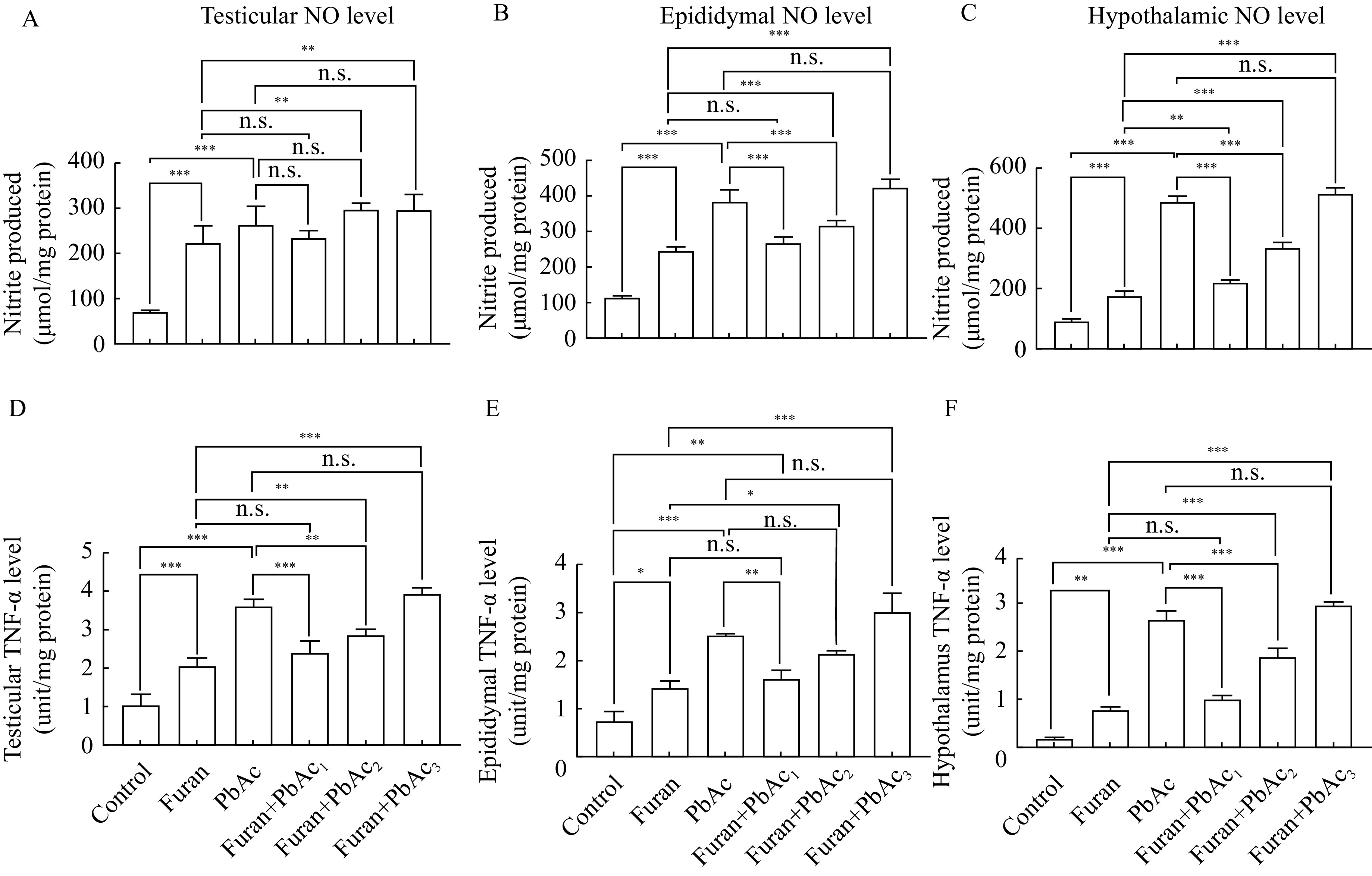
Lead and furan treatments increased the levels of NO and TNF-α in male Albino Wistar rats.

**Figure 11:**
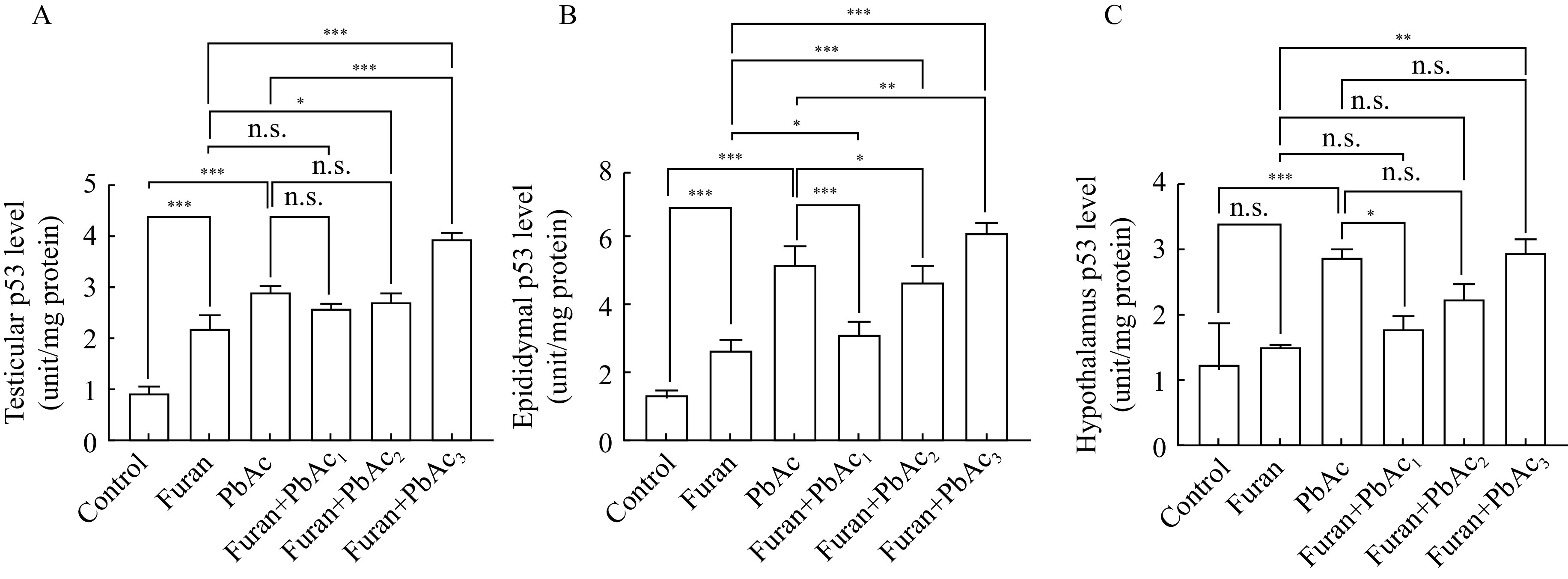
Lead and furan treatments increased the levels of p53 in male Albino Wistar rats.

Excessive oxidative stress could induce inflammation and genomic instability if not minimized. In this study, we examined the effects of PbAc and furan on pro-inflammatory mediators and p53 levels in rats' hypothalamus, testis, and epididymis. The results showed that PbAc and furan mitigated tissue homeostasis by significantly increasing (*P<*0.05) the levels of NO and TNF-α in the hypothalamus, testis, and epididymis of rats compared to the control group (***Fig. 10***). In addition, PbAc and furan markedly raised the tissue levels of p53 in the testis, epididymis, and hypothalamus, indicating the potential of cell cycle arrest, DNA repair and/or apoptosis (***Fig. 11***). This indicates that treatments with PbAc and furan orchestrated inflammation and genomic instability in the hypothalamus, testis, and epididymis of rats.

### Effect of combined treatment of Pb and furan on the histological features of the testis and epididymis of rats

**Figure 12:**
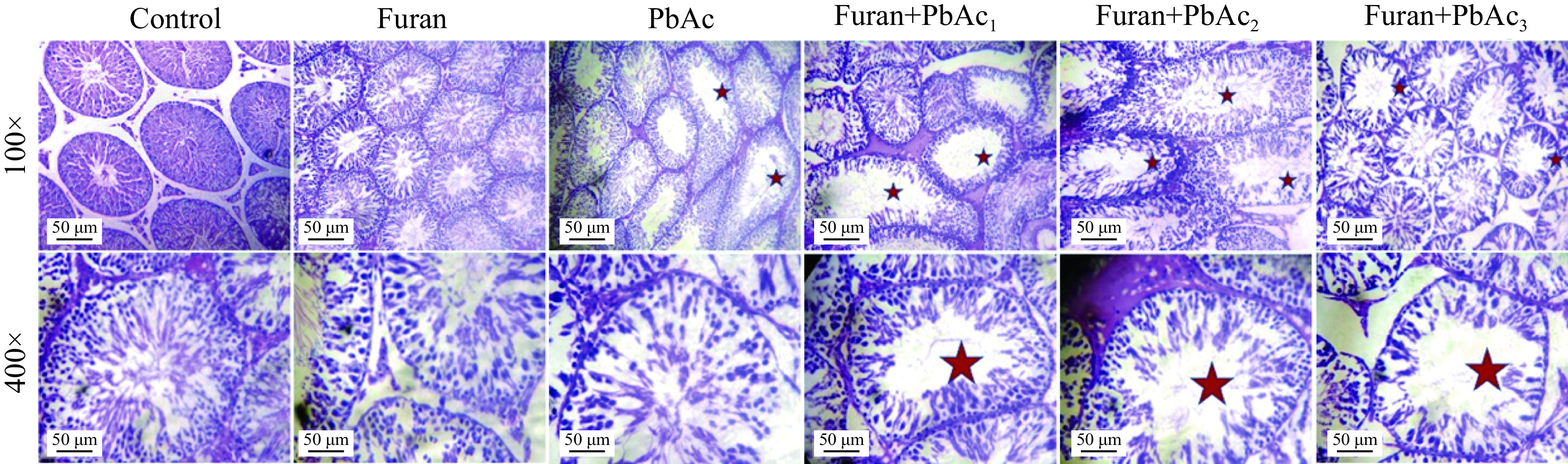
Histology of the representative photomicrographs of the experimental Albino Wistar rats' testes stained with haematoxylin and eosin.

Histology of the testes stained with haematoxylin and eosin at two different magnifications (100× top row; 400× bottom row) are shown in ***Fig. 12***. Rats in control cohorts exhibited typical testicular architecture, seminiferous tubules, lumen replete in spermatozoa, typical Sertoli, Leydig and spermatogonia cells within the interstitial spaces, while cohorts of rats treated with PbAc alone showed interstitial erosion of the testicular lumen. In addition, the spermatozoa number present in the lumen of furan alone treated experimental rats was gradually reduced relative to the control cohort. The observed testicular deterioration and reduction of sperm number in rat were worsened as the doses of PbAc was increased at a constant dose of furan. These outcomes indicated that PbAc and furan treatments impaired the histoarchitectural features of the testis and epididymis of rats.

## Discussion

We investigated whether combined exposure to Pb and furan synergistically exacerbate oxidative stress, inflammation, and genomic stress in rat hypothalamic-pituitary-testicular system. The alteration in mean body weight and organ to body weight ratio indicates the onset of tissue dysfunction emanating from Pb and furan exposure may be associated with ROS and RNS accumulation. Increased RONS can trigger cell death if left unchecked^[40]^. Specifically, the infiltration of cytokines in tissues may result in a cytokine storm, which in turn stimulates the hypothalamic-pituitary-adrenal axis to release adrenocorticotropic hormones (ACTH) and subsequently glucocorticoids. Glucocorticoids promote gluconeogenesis, amino acid mobilisation, and fat breakdown. These macromolecules are needed to maintain the size and weight of the examined organs, and their utilisation to abate Pb and furan stress may lead to tissue atrophy observed in our and others' studies ^[17]^.

The hypothalamic-pituitary-testicular system is tightly regulated, and the functions of the male reproductive system depend on the maintenance of the level of gonadotropin-releasing hormones (GnRH) including LH and FSH. LH is a glycoprotein that stimulates testicular Leydig cells to synthesise testosterone and control spermatogenesis. In addition, FSH rouses sperm production in the Sertoli cells. Prolactin is known to exert a short loop of negative feedback on LH and FSH in oxidative and inflammatory stress. Reduction in LH and FSH levels adversely reduces spermatogenesis and the activities of testicular enzymes^[2]^. Co-exposure to Pb and furan suppressed LH, FSH, and testosterone production, while prolactin level increased. This inverse correlation may have been brought about by the action of ROS and pro-inflammatory mediators on the hypothalamic-pituitary-testicular system. Accumulation of ROS and pro-inflammatory cytokines induces ACTH expression that drives glucocorticoid production. Glucocorticoid negatively influences GnRHs and dysregulates the biochemical and physiological processes that drive spermatogenesis and steroidogenesis. The aftermaths of such negative regulation include increased sperm abnormality observed in this study corroborating previous report^[12]^. A limitation of this study is a lack of data on GnRH and its antagonist gonadotropin-inhibitory hormone (GnIH), which plays an active role in reproductive function^[41]^. GnRH regulates FSH and LH release from the anterior pituitary enhancing reproductive effectiveness. These essential sex regulatory/inhibitory hormones from the gonads are regulated *via* the hypothalamus-pituitary-gonadal (HPG) axis^[42]^ and experimental data can be insightful to reproductive function.

Furthermore, the integrity of these cells to drive spermatogenesis depends testicular ACP, ALP, G6PD and LDH activities. ACP provides phosphate during the development, and maturation of testicular Sertoli and Leydig cells^[43]^, and the observed reduction in these enzyme activities indicate a defect in rat reproductive status. Also, LDH is widely expressed in the testis, and drives several processes necessary for male fertility and sperm function. For instance, LDH activity furnishes the Embden Meyerhof-Parnas pathway with NAD^+^ needed to sustain the processes of glycolysis and ATP production in the flagellum. In addition, G6PD in the hexose monophosphate shunt generates NADPH -a reducing equivalent- and the sugar moiety (ribose 5-phosphate) to maintain spermatogenesis and steroidogenesis^[44]^. While ALP expressed in the Leydig cells and SSCs of the testis regulate several metabolic activities during spermatogenesis and testicular steroidogenesis^[45]^. Decreases in these enzyme activities indicate testicular atrophy, degeneration of the seminiferous epithelium, and dysfunction in the hypothalamic-pituitary-testicular system. Therefore, we validate the hypothesis that reduced reproductive health of male rats is associated with the suppression of ACP, ALP, G6PD, and LDH activities.

We observed that animals co-exposed to Pb and furan exhibited a significant reduction in the GSH and TSH levels and the activities of antioxidant enzymes, such as CAT, SOD, GST, and GPx. This observation implies inadequate antioxidants necessary to scavenge harmful free radicals and prevent the induction of oxidative stress in the examined tissues. The increased levels of LPO and RONS and the activities of XO and MPO in the examined tissues of rats co-exposed to Pb and furan occasioned RONS production that may overwhelm the antioxidant capability. Consequently, the exposure led to oxidative damage in the testes, epididymis, and hypothalamus. ROS mediates toxicity by inducing lipid peroxidation, formation of protein crosslinks, and oxidative DNA damage. These metabolic processes invariably inhibit essential processes involved in spermatogenesis and steroidogenesis, reducing sperm quality and male fertility^[40]^.

Overproduction of NO induces nitrosative stress, which reportedly modifies cellular proteins, lipids, and nucleic acids after exhausting antioxidant defence systems^[46]^. Also, increased TNF-α levels in the hypothalamus, testis and epididymis of rats are indicative of tissue injury. TNF-α in the company of other pro-inflammatory cytokines such as IL-1β, IL-6, and IL-8 stimulate hypertrophy of the Sertoli and Leydig cells as well as the infiltration of pro-inflammatory cells into the spermatocytes and spermatids. In addition, the accumulation of ROS and pro-inflammatory mediators may trigger loss of genomic integrity by oxidative DNA damage. Excessive inflammation and oxidative stress are associated with activating p53 to regulate important cellular and molecular decisions, including cell cycle arrest, DNA repair, and apoptosis. We observed that Pb and furan treatment triggered the activation of rat p53 in the hypothalamus, testis, and epididymis that may have occurred to avert defective DNA replication into the spermatogonial nuclei during spermatogenesis. Histopathology findings support the altered morphology, tissue histoarchitectural defect and functionality caused by Pb and furan treatment. Treatment with Pb and furan increasingly exacerbated tissue damage in the co-treated groups stemming from increased Pb concentration in the drinking water. In addition, Pb exposure is well established to be toxic *via* several mechanisms on reproductive dysfunction^[47]^.

**Figure 13:**
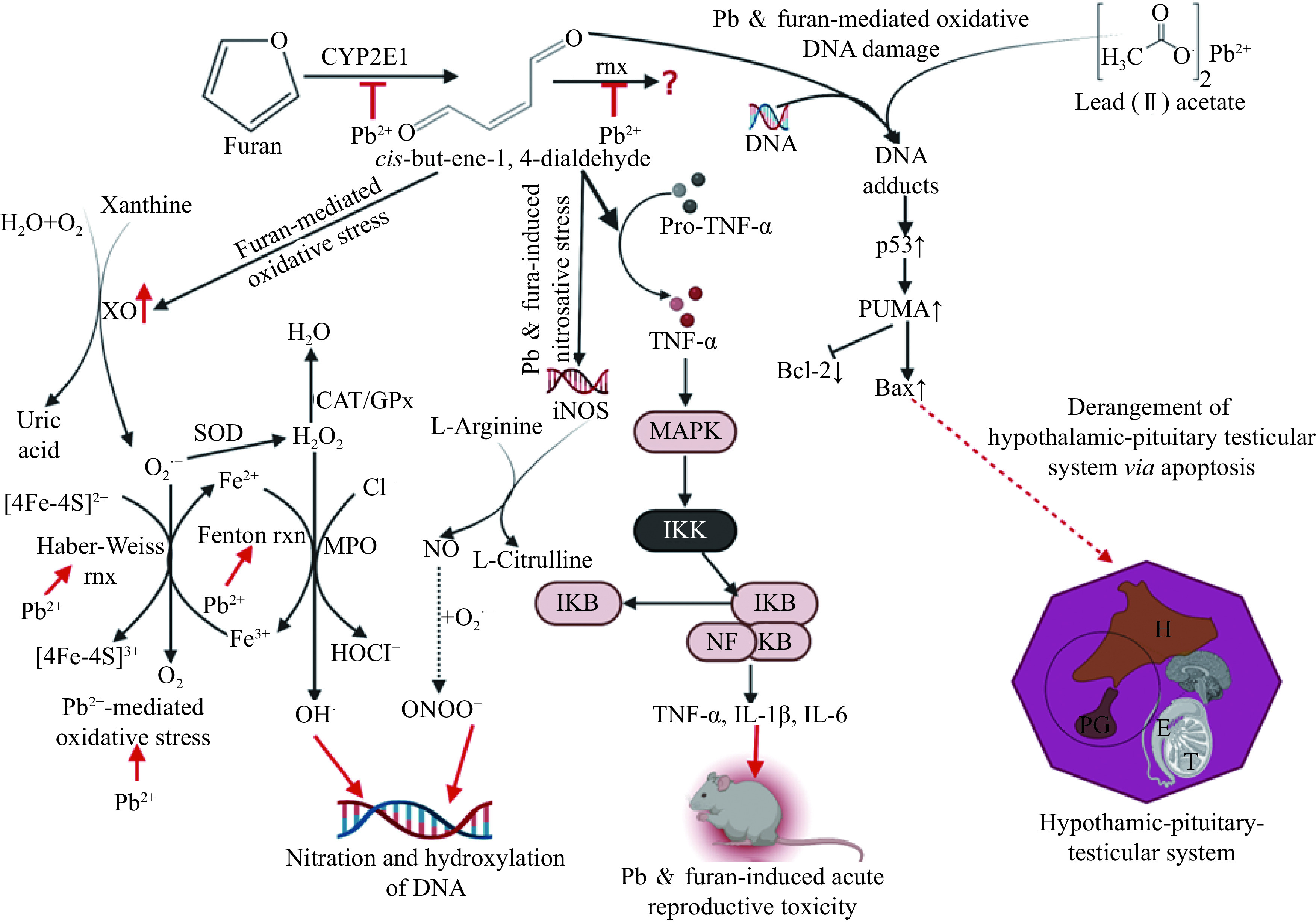
Proposed mechanisms of inadvertent co-exposure of experimental rat models to Lead and furan in diet and water, respectively.

Taken together, our study shows that co-exposure to Pb and furan altered the integrity of the hypothalamic-pituitary-testicular system *via* the induction of oxidative stress, inflammation, and loss of genomic stability. The probable mechanisms of toxicity of Pb and furan include altering signalling networks including extracellular signal-regulated kinase, nuclear factor kappa B and the repression of signalling networks including nuclear factor erythroid 2-related factor-2^[48]^ as depicted in our proposed toxicity mechanism (***Fig. 13***). More stringent measures should be taken to regulate indiscriminate anthropogenic activities and monitor food processing methods that are known to exacerbate Pb and furan toxicity. Moreover, such inadvertent exposure also have damaging effect on neurobehavioral-coordination as outlined in our latest findings^[49]^.
